# Relative importance of redox buffers GSH and NAD(P)H in age-related neurodegeneration and Alzheimer disease-like mouse neurons

**DOI:** 10.1111/acel.12216

**Published:** 2014-03-21

**Authors:** Debolina Ghosh, Kelsey R Levault, Gregory J Brewer

**Affiliations:** 1 Department of Medical Microbiology, Immunology and Cell Biology, Southern Illinois University School of MedicineSpringfield, IL, 62794-9626, USA; 2 Department of Neurology, Southern Illinois University School of MedicineSpringfield, IL, 62794-9626, USA; 3 Department of Biomedical Engineering, University of CaliforniaIrvine, CA, 92697-2715, USA

**Keywords:** 3xTg-AD, aging, glutathione, NAD(P)H, neurodegeneration, redox

## Abstract

Aging, a major risk factor in Alzheimer’s disease (AD), is associated with an oxidative redox shift, decreased redox buffer protection, and increased free radical reactive oxygen species (ROS) generation, probably linked to mitochondrial dysfunction. While NADH is the ultimate electron donor for many redox reactions, including oxidative phosphorylation, glutathione (GSH) is the major ROS detoxifying redox buffer in the cell. Here, we explored the relative importance of NADH and GSH to neurodegeneration in aging and AD neurons from nontransgenic and 3xTg-AD mice by inhibiting their synthesis to determine whether NADH can compensate for the GSH loss to maintain redox balance. Neurons stressed by either depleting NAD(P)H or GSH indicated that NADH redox control is upstream of GSH levels. Further, although depletion of NAD(P)H or GSH correlated linearly with neuron death, compared with GSH depletion, higher neurodegeneration was observed when NAD(P)H was extrapolated to zero, especially in old age, and in the 3xTg-AD neurons. We also observed an age-dependent loss of gene expression of key redox-dependent biosynthetic enzymes, NAMPT (nicotinamide phosphoribosyltransferase), and NNT (nicotinamide nucleotide transhydrogenase). Moreover, age-related correlations between brain NNT or NAMPT gene expression and NADPH levels suggest that these genes contribute to the age-related declines in NAD(P)H. Our data indicate that in aging and more so in AD-like neurons, NAD(P)H redox control is upstream of GSH and an oxidative redox shift that promotes neurodegeneration. Thus, NAD(P)H generation may be a more efficacious therapeutic target upstream of GSH and ROS.

## Introduction

While the major electron transfer currencies for oxidation and reduction reactions in cells are the redox couples NADH/NAD^+^ and NADPH/NADP^+^, glutathione (GSH) at 1–2 mm is the major redox buffer in neurons (Johnson *et al*., 2012) and other cells. In the brain, NADH is the major electron donor to power the mitochondrial electron transport chain for ATP synthesis. There are several pathways for NADH generation including glycolysis, Kreb’s cycle dehydrogenases, and the NAMPT-dependent salvage pathway. While two molecules of NADH are generated in glycolysis, the Kreb’s cycle dehydrogenases including pyruvate dehydrogenase, isocytrate dehydrogenase, α-ketogluterate dehydrogenase, and malate dehydrogenase synthesize NADH and feed most of the NADH required for the electron transport chain. The salvage pathway is needed to regenerate NAD^+^ from the nicotinamide produced by sirtuin and poly (ADP-ribose) polymerase (PARP) consumption via the rate-limiting enzyme nicotinamide phosphoribosyltransferase (NAMPT). Under energetic demand, mitochondrial NADH can also be regenerated through the inner mitochondrial membrane-resident nicotinamide nucleotide transhydrogenase (Nnt) (Yin *et al*., 2012). NAMPT appears to contribute to replicative senescence as van der Veer *et al*. (2007) showed that overexpression of NAMPT restored replication in human smooth muscle cells. Moreover, in the presence of nicotinamide, NAMPT can delay axonal degeneration *in vitro* (Sasaki *et al*., 2006). Thus, a decline of NAMPT in aging or age-associated Alzheimer’s disease (AD) brain may decrease NADH levels that in turn could cause an oxidized redox shift, lower GSH levels and promote neurodegeneration (Brewer, 2010; Ghosh *et al*., 2012).

Although NADH can be indirectly generated from GSH (Icen, 1967), it is not clear whether NADH levels depend more on GSH or GSH levels reflect NADH levels under conditions of oxidative/redox stress and neurodegeneration. Glutathione is either synthesized by conjugation of glutamate to cysteine by the rate-limiting glutathione cysteine ligase (GCL) (with subsequent addition of glycine by glutathione synthase) or regenerated from the oxidized form, GSSG by glutathione reductase (GR). GR requires NADPH as a substrate, and thus, NADPH is directly linked to GSH synthesis. Nnt catalyzes mitochondrial transmembrane hydride transfer between NAD(H) and NAD(P)^+^ to generate NAD(P)H. In mouse brain, Nnt catalyzed NADPH generation contributes to about 50% of the total mitochondrial NADPH pool (Rydstrom, 2006). This NADPH donates electrons to regenerate GSH from GSSG. Thus, deficits in Nnt gene expression, or redox-sensitive activity or availability of substrates may impact NADPH and GSH synthesis and cause a shift to an oxidized redox state that is deleterious to redox-sensitive transporters, transcription factors, and even neuron viability. Further, to determine the best target for slowing neurodegeneration, clarity is needed about whether kinetics and compartmentalization within the cell (Jones & Go, 2010) position either NAD(P)H or GSH upstream, in disequilibrium.

We recently observed that in neurons from aging mice, both NADH regenerating capacity and GSH levels declined and ROS levels increased (Ghosh *et al*., 2012). Moreover, in AD-like neurons from a transgenic mouse, both NADH regenerating capacity and GSH levels were lower than those in non-Tg neurons from 2 months of age. In titrating GSH levels by inhibiting glutathione cysteine ligase in cultured mouse neurons, we also observed that GSH depletion is more important than ROS elevation in causing increased neruon death in aging and AD neurons (Ghosh *et al*., 2014). To further establish this conclusion, here we evaluate whether inhibition of GSH or NADH synthesis is more vital for maintaining redox balance and viability in the neuron. Moreover, under stress with limited availibility of GSH, it is not known whether NADH can compensate for the GSH loss to maintain redox balance or whether NADH declines as well. Here, we report the relative importance and interdependency of these two major redox buffers, NAD(P)H and GSH in aging and AD-like mouse model neurons.

We used neurons isolated and cultured from LaFerla’s triple transgenic mouse model of AD (3xTg-AD) and nontransgenic mice (non-Tg) (Oddo *et al*., 2003) across the lifespan (Ghosh *et al*., 2012) to separate age-related intrinsic changes in neurons from an aging hormone, immune, and vascular system (Brewer & Torricelli, 2007). This model has human mutant amyloid precursor protein (*APP*), presenilin 1 (*PS1*), and tau that lead to increased deposition of A-beta and memory deficits by 6 months, although in our colony, memory deficits in males were not detected until 18–22 months. We determined the redox shift in these cultured neurons as intrinsic fluorescence of intracellular NAD(P)H (Chance *et al*., 1979). As intrinsic fluorescence cannot distinguish between NADH and NADPH, the more general term NAD(P)H is used. However, 80% of autofluorescence originates from NADH in cardiac myocytes (Eng *et al*., 1989). Here, the overall aim was to determine which is more important in aging and Alzheimer’s disease, NAD(P)H or glutathione, by titrated inhibition of GSH synthesis with buthionine sulfoximine (BSO) (Hulbert & Yakubu, 1983) or NAD(P)H synthesis with FK866 (Wang *et al*., 2011). We report that in aging neurons and 3xTg-AD neurons, NAD(P)H depletion is more seminal for neurodegeneration than GSH.

## Results

### NAMPT inhibition decreases NAD(P)H in both non-Tg and 3xTg-AD neurons

**Figure 1:**
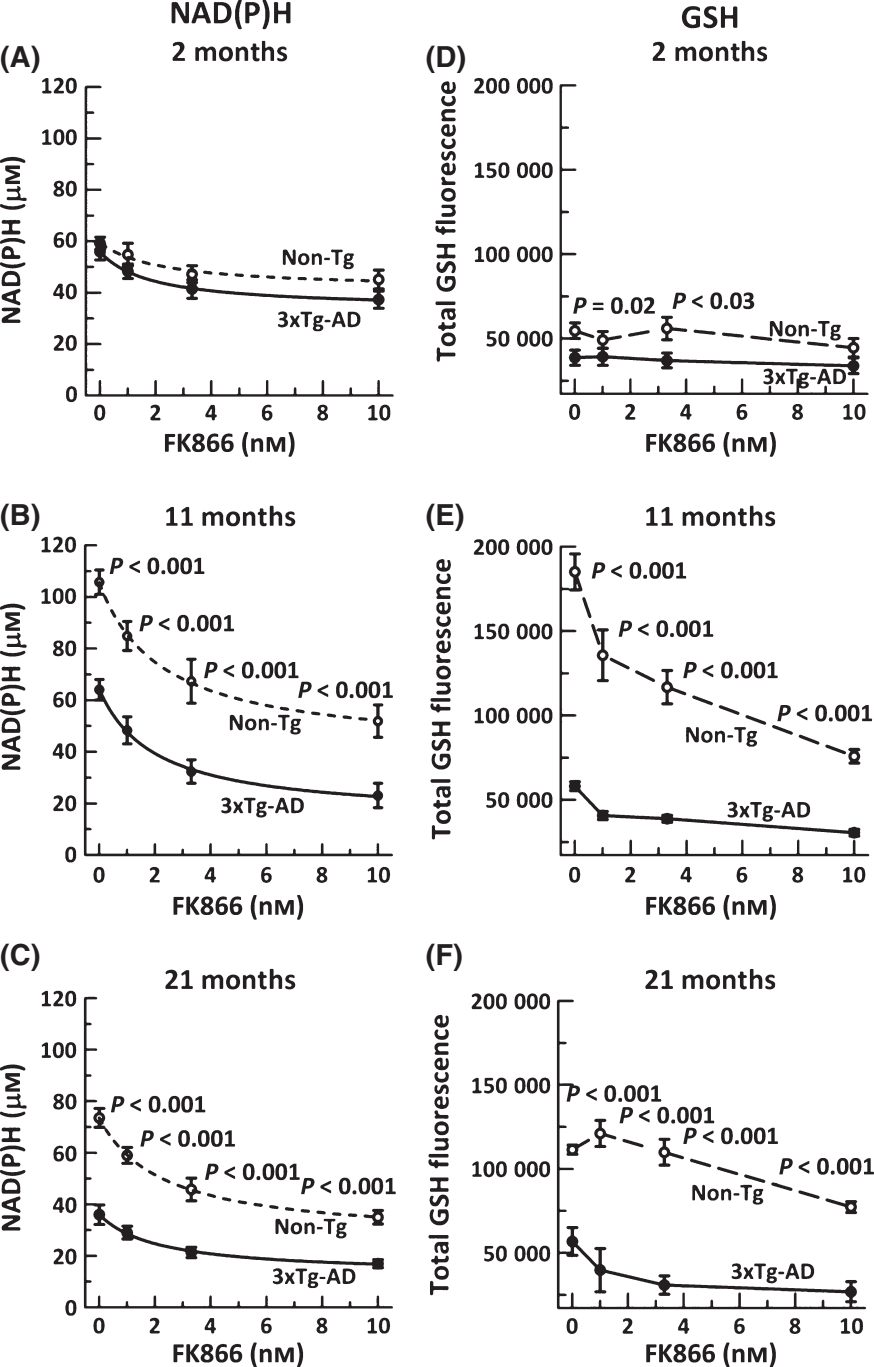
Inhibition of NAMPT decreases NAD(P)H and glutathione levels in both non-Tg and 3xTg-AD neurons. NAMPT inhibitory doses of FK866 decreased NAD(P)H levels in non-Tg (open circle, dashed line) and 3xTg-AD (filled circles, solid line) neurons in A) 2-month (ANOVA genotype F(1,116) = 4.3, *P* = 0.04, FK866 F(3,116) = 6.9, *P* < 0.001, B) 11-month (ANOVA genotype F(1,59) = 82, *P* < 0.001, FK866 F(3,59) = 26, *P* < 0.001), and C) 21-month (ANOVA genotype F(1,108) = 149, *P* < 0.001, FK866 F(3,108) = 31, *P* < 0.001) mice. *n* = 15–20 neurons from 3–4 mice per age per genotype. Effects of the same dose-dependent inhibition of NAMPT on GSH levels were small at D) 2 months, (ANOVA genotype F(1,56) = 11, *P* = 0.001, FK866 F(3,56) = 0.7, *P* = 0.57), but significantly decreased glutathione at E) 11 months (ANOVA genotype F(1,112) = 72, *P* < 0.001, FK866 F(3,112) = 46, *P* < 0.001), and F) 21 months (ANOVA genotype F(1,117) = 28, *P* < 0.001, FK866 F(3,117) = 128, *P* < 0.001) in non-Tg (open circle, dashed lines) or 3xTg-AD (filled circle, solid line). *n* > 350 neurons from 3–4 mice per age per genotype.

Nicotinamide phosphoribosyltransferase (NAMPT) is the rate-limiting enzyme for NAD^+^ synthesis via the salvage pathway, while transhydrogenase and Kreb’s cycle dehydrogenases convert NAD^+^ to reduced NADH. If NAD^+^ is merely recycled without consumption, then inhibition of NAMPT will have no effect on NAD(P)H levels. However, if sirtuins and PARPs consume NAD^+^ to produce nicotinamide and O-acetyl-ADP-ribose, then replenishment of the NADH/NAD^+^ pool will require resynthesis through nicotinamide by NAMPT. To determine the importance of NAMPT to maintain NADH levels, we treated neurons for 15 h with different concentrations of the specific NAMPT inhibitor FK866 (Wang *et al*., 2011). In young 2-month non-Tg neurons (Fig. 1A), NAMPT inhibition decreased NAD(P)H by 24% from 59 to 45 μm at the highest 10 nm FK866 concentration, while 3xTg-AD neurons were inhibited 34% from 56 to 37 μm. These small decreases in young 2-month neurons suggest that most of the neuronal NAD^+^ is conserved and recycled through transhydrogenase and dehydrogenases. In 11-month neurons on the other hand (Fig. 1B), NAD(P)H levels start higher at 106 and 64 μm for non-Tg and 3xTg-AD neurons, respectively, with larger maximal inhibition of 51% to 52 μm and 64% to 23 μm. Similarly, large effects were observed in 21-month neurons (Fig. 1C), with maximal 53% inhibition of NAD(P)H levels to 35 μm for non-Tg and 50% to 17 μm for 3xTg-AD neurons. These large losses indicate greater consumption of the NAD^+^/NAD(P)H pool in 11- and 21-month than 2-month neurons. The increase in starting levels of NAD(P)H in middle age followed by the decline in old age indicates the importance of NAD(P)H for aging, as previously described and discussed by us in Ghosh *et al*. (2012). The larger genotype differences suggest increased consumption of NAD^+^ or impaired resynthesis in the 3xTg-AD neurons (Liu *et al*., 2009). Of final note, the IC50s in each case were about 1.5 μm FK866, indicating that neither age nor genotype affected the mechanism of inhibition. In summary, FK866 inhibition of NAMPT has large effects on NAD(P)H levels starting at middle age, suggesting neuron reliance on resynthesis through the salvage pathway. In practice, increases in inhibition of NAMPT by collective increments in FK866 increasingly depletes NAD(P)H in both genotypes which enabled its use in determination of the effects of NAD(P)H depletion on GSH levels and neurodegeneration.

### Decreasing NAD(P)H levels decreased glutathione levels in aging and AD-like neurons beginning at middle age

GSH redox regeneration from GSSG depends on NADPH via glutathione reductase (Kosower & Kosower, 1978) and nicotinamide nucleotide transhydrogenase (NNT) for transformation of NADH to NADPH (Olgun A, 2009), however, whether NADH and GSH redox systems are interdependent or one is upstream of the other is not well studied in whole cells. As autofluorescence from NAD(P)H is ~80% NADH (Eng *et al*., 1989), we can assume that our measures are more indicative of NADH fluorescence, but could be highly dependent on transhydrogenase to maintain sufficient levels of NADPH. To determine whether depleting NAD(P)H has an effect on glutathione levels or whether they are independent with compensation for GSH oxidation and loss by *de novo* synthesis, we stressed neurons with the NAMPT inhibitor FK866 and measured glutathione levels in individual live neurons. We hypothesized that if NAD(P)H redox control is upstream of GSH redox control, then depleting NAD(P)H will also deplete GSH levels. Although at 2 months, glutathione levels were largely independent of NAD(P)H depletion (Fig. 1D, ANOVA (FK866), *P* = 0.568), beyond middle age, there was a dramatic effect of NAD(P)H depletion on GSH levels for both the genotypes. In non-Tg neurons, at 11 and 21 months (Fig. 1 E,F), a stress of 10 nm FK866 resulted in 59% and 31% loss of glutathione, respectively, compared with unstressed neurons. The 3xTg-AD neurons on the other hand were more sensitive to NAD(P)H depletion with 47% and 53% loss of GSH for neurons from 11- and 21-month brains (Fig. 1 E,F), but these lower levels of GSH were highly influenced by dramatically lower starting levels (Ghosh *et al*., 2012). Overall, the depletion of NAD(P)H depleted GSH suggesting that NAD(P)H redox control is upstream of GSH regeneration or in close equilibrium.

### NAD(P)H depletion leads to increased cell death in non-Tg and 3xTg-AD neurons

**Figure 2:**
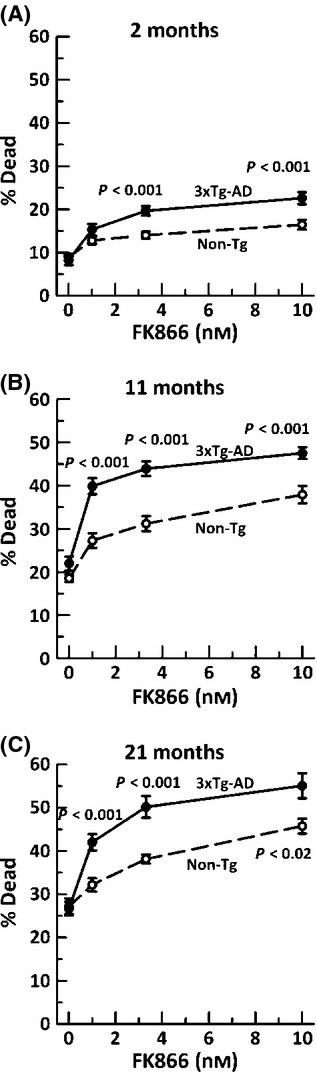
Increased neuron death with age subsequent to NAD(P)H depletion in both non-Tg (black open circle, dashed lines) and 3xTg-AD (black filled circle, solid line) neurons from A) 2-month mice, (ANOVA genotype F(1,120) = 19, *P* < 0.001, FK866 F(3,120) = 37, *P* < 0.001), B) 11-month mice, (ANOVA genotype F(1,129) = 42, *P* < 0.001, FK866 F(3,129) = 73, *P* < 0.001), or C) 21-month mice, (ANOVA genotype F(1,76) = 34, *P* < 0.001, FK866 F(3,76) = 53, *P* < 0.001). *n* = 15–20 neurons from 3–4 animals per age and genotype.

As NAD(P)H is vital for maintaining the redox state for viability, we wanted to examine the effect of NAD(P)H depletion on neurodegeneration. Although the percent dead cells were similar in both genotypes under the unstressed condition (Fig. 2), inhibiting NAMPT gradually increased the cell loss by 1.5-fold in non-Tg neurons and a significantly higher twofold in 3xTg-AD neurons at all ages. There was also an age-related increase in susceptibility of neurons of both genotypes to NAMPT inhibition, suggesting greater dependence of viability under stress on NAD^+^ in old neurons.

### Linear correlations of neurodegeneration and GSH dependent on NAD(P)H levels

HPLC analysis of brain thiols (Table 1) indicated that 21-month 3xTg-AD brain GSH, GSH/GSSG, and redox state were significantly more oxidized than non-Tg brains (lower GSH, lower ratio, and less negative redox state). In comparison with other aging brain measures (Table 1), our results generally agree with an age-related oxidative shift, but numerical values vary greatly with genotype; strain; the method used for isolation, derivatization, and measurement. As the brain consists of neurons, glia, and endothelial cells, all buffered by the blood, whole brain homogenates are unlikely to reflect the *in vivo* status of the neurons themselves. Here, we focused on monochlorobimane derivatives of GSH measured by fluorescence detection in single live neurons (Kamencic *et al*., 2000; Ghosh *et al*., 2012), as quantities of these neurons are insufficient for detection by HPLC.

**Table 1:** Brain GSH and GSSG measures (nmol/mg brain)

		GSH (±SE)		GSSG (±SE)		Redox ratio		Redox state (mV)		Reference
Animals	Method	Young	Old	Young	Old	Young	Old	Young	Old	
Sprague–Dawley rats (15–18, 29–30 month. female) (*n* = 6–9)	DTNB deriv. colorimetric	1.47 (0.25)	0.85 (0.56)**	N/A		N/A				Ravindranath *et al*., 1989;
Fisher rats (3, 26 month.) *n* = 15	DTNB deriv. HPLC-UV	1.7 (0.2)	1.1 (0.1)*	0.06 (0.01)	0.08 (0.01)	28 (4)	14 (1)**			Suh *et al*., 2004;
Sprague–Dawley rats (4,17 month.) male/female (*n* = 5)	DTNB deriv. colorimetric	0.98/1.1	0.83 **/0.83 *	0.020/0.22	0.026 **0.27*	49/43	35**/36*			Zhu *et al*., 2006;
C57BL/6 (3, 21 month.) male *n* = 30	HPLC-colorimetric EC	17	12.5 *	0.08	0.12 *	200	100*			Rebrin *et al*., 2007;
DBA/2, (3, 21 month) male *n* = 25	HPLC-colorimetric EC	16	12.5 *	0.12	0.14	128	92*			Rebrin *et al*., 2007;
C57BL6/129 3xTg-AD (2, 21 month.) male, *n* = 10–13	HPLC-fluorometric	0.94 (0.02)	0.92 (0.02)	0.121 (0.005)	0.116 (0.004)	8.1 (0.3)	8.0 (0.3)	−170.1 (0.8)	−169.4 (1.5)	G hosh *et al*., 2014; present article
		0.92 (0.02)	0.82 (0.03)*	0.115 (0.003)	0.119 (0.003)	7.5 (0.5)	7.0 (0.3)*	−170.0 (0.7)	−166.6 (1.1)*	
		*P = 0.01 old genotype				*P = 0.01 old genotype		*P = 0.01 old genotype		

*P < 0.05, **P < 0.001, N/A not available, DTNB, 1-fluoro-2,4-dinitrobenzene.

**Figure 3:**
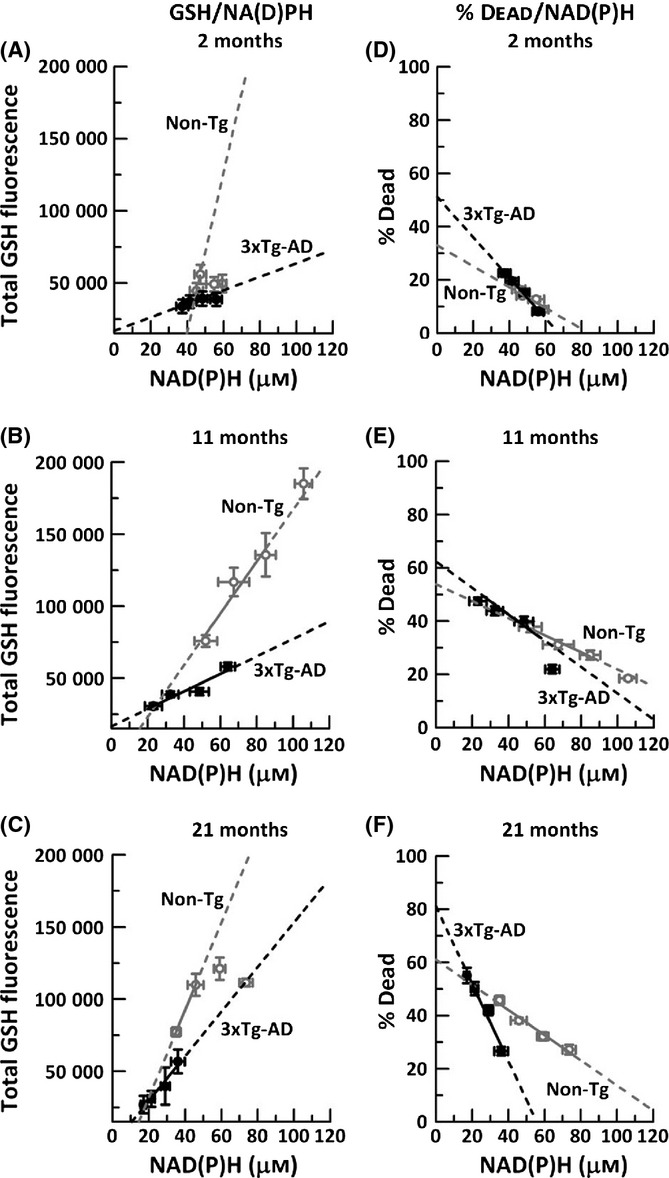
GSH levels and neurodegeneration are highly dependent on NAD(P)H levels. A–C, GSH dependence with controlled decrements in NAD(P)H; D–F, effects of these decrements on neuron death. Extrapolation of non-Tg (gray open circles, dashed lines) and 3xTg-AD to zero GSH in A) 2-month neurons (R^2^ for non-Tg = 0.82, 3xTg-AD = 0.83), B) 11-month neurons (R^2^ for non-Tg = 0.94, 3xTg-AD = 0.9), and C) 21-month neurons (R^2^ for non-Tg = 0.734, 3xTg-AD = 0.95). Note that linear fits for 2-month and 21-month non-Tg neurons excluded several high GSH values because they were unaffected by decrements in NAD(P)H. Similarly, neurodegeneration increases with decrements in NAD(P)H in both genotypes, in D) 2-month neurons (non-Tg slope = −0.44;R^2^ = 0.89, 3xTg-AD slope = −0.76; R^2^ = 0.99), E) 11-month neurons (non-Tg slope = −0.35; R^2^ = 0.99, 3xTg-AD slope = −0.59; R^2^ = 0.89, and F) 21-month (non-Tg slope = −0.47; R^2^ = 0.97, 3xTg-AD slope = −1.46; R^2^ = 0.97). *n* = 3–4 animals per genotype per age.

We determined whether redox control of NAD(P)H is upstream of GSH and neurodegeneration by plotting a) GSH levels as a function of NAD(P)H depletion (Fig. 3A–C) and b) neurodegeneration as a function of NAD(P)H depletion (Fig. 3D–F) with extrapolation to either zero NAD(P)H or zero GSH. Note that the GSH scale is plotted to begin at our threshold of detection, which we consider zero GSH (about 23000 density x area units). GSH levels are proportional to our measures of monochlorobimane fluorescence (Kamencic *et al*., 2000), which is further supported in our system by HPLC measures of brain GSH and GSSG (Ghosh *et al*., 2014). In neurons, all ages showed large positive slopes with excellent linear fits (Fig. 3A–C), supporting the known relationship of GSSG reduction to GSH by glutathione reductase with NADPH. Importantly, viable non-Tg and 21 month 3xT-AD neurons extrapolated to non-zero NAD(P)H as GSH levels reached zero, suggesting that NAD(P)H is upstream of GSH and that NAD(P)H is essential for viability. The slopes for the non-Tg neurons were steeper than those for 3xT-AD neurons, suggesting impairment in the coupling of NAD(P)H to GSH in the 3x-Tg neurons at all ages, possibly explained by lower starting levels and an oxidized redox state for NAD(P)H in 3xTg-AD neurons. Together, these results suggest that NAD(P)H levels are upstream and control GSH levels in both an age- and genotype-dependent fashion.

Simultaneously, we determined the impact of these forced deficits in NAD(P)H and resultant decreases in GSH on neurodegeneration (Fig. 3D–F). Further, we examine the relative contributions of GSH and NAD(P)H depletion to neurodegeneration. Neurodegeneration is the process that ultimately leads to neuronal death which we measure here as loss of plasma membrane integrity that allows propidium iodide to enter the neuron and stain nuclear DNA. Decreased NAD(P)H concentrations by FK866 inhibition of NAMPT led to 2- to 3-fold increases in percent death in both non-Tg and 3xTg-AD neurons at all ages. Moreover, the slope of %dead/NAD(P)H for non-Tg neurons remained approximately the same with age, indicating that neuron loss increases similarly with NAD(P)H depletion at all ages. On the other hand, 3xTg-AD neurons were more sensitive to NAD(P)H depletion from the earliest 2 month age and continued to show strong negative correlation of % dead with NAD(P)H loss until the oldest 21 months. Extrapolating to zero μm NAD(P)H led to 32% non-Tg neuron loss, while the 3xTg-AD neurons were 1.5-fold higher at 50%, indicating that 3xTg-AD neurons are more vulnerable to NAD(P)H loss. With aging, the % dead increased even further in both genotypes, and at 21 month, we observed a 60% cell death for non-Tg neurons, while 3xTg-AD neurons had a dramatic 80% neuron loss, 1.3-fold higher than non-Tg neurons. Overall, these results indicate a direct dependence of neurodegeneration on NAD(P)H availability. Further, the susceptibility to neurodegeneration by NAD(P)H loss increases with age in both genotypes, but is more severe in the 3xTg-AD neurons.

### NAD(P)H levels in 3xTg-AD neurons decline with GSH loss at all ages

**Figure 4:**
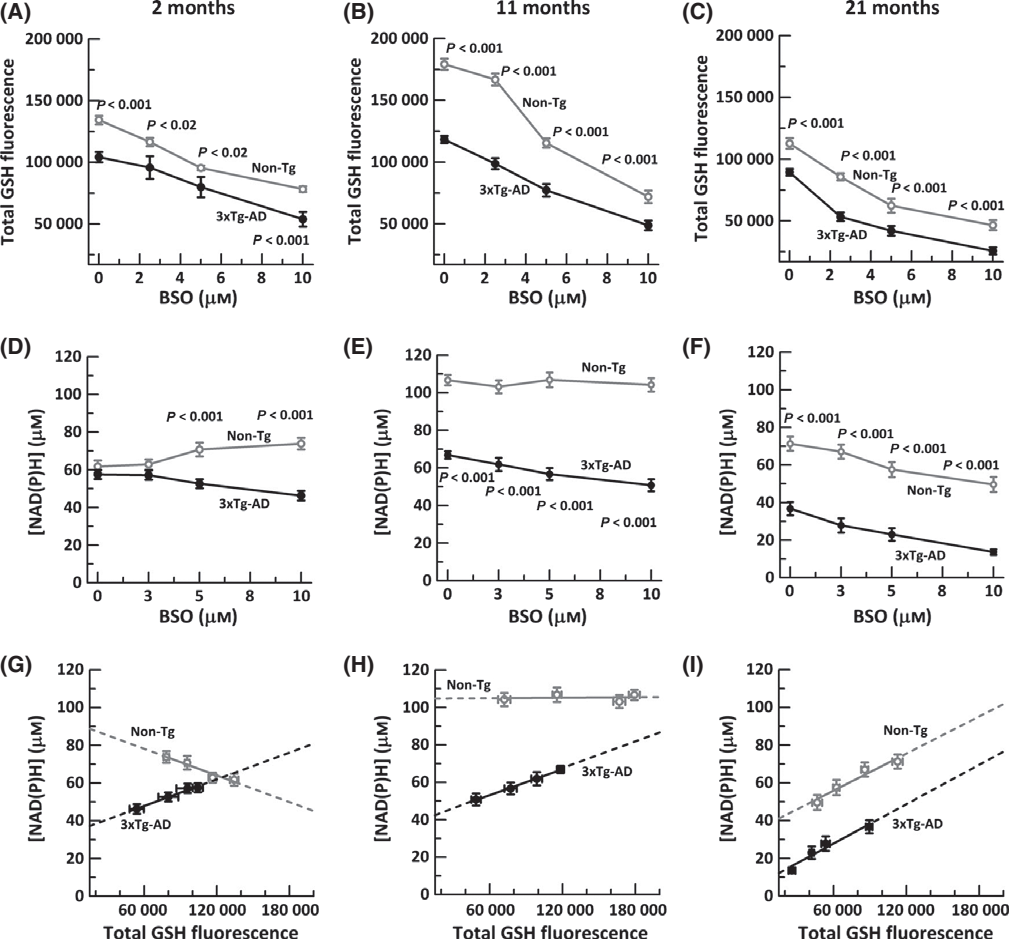
Inhibition of GSH synthesis gradually alters NAD(P)H levels depending on age with decrements in the 3xTg-AD neurons similar to old non-Tg neurons. A) Titration of glutathione levels with indicated BSO concentrations in non-Tg (gray open circle, gray solid line) and 3xTg-AD mice (black filled circle, black solid line) in 2-month (ANOVA genotype F(1,104) = 9.9, *P* < 0.03, BSO F(3,104) = 48, *P* < 0.001), B)11-month neurons (ANOVA genotype F(1,119) = 128, *P* < 0.001, BSO F(3,119) = 89, *P* < 0. 001), and C) 21-month neurons (ANOVA genotype F(1,109) = 37, *P* < 0.001, BSO F(3,109) = 54, *P* < 0. 001). *n* = 250–300 neurons from 3–4 animals per age per genotype. Neuronal NAD(P)H levels in non-Tg neurons remain unaffected by GSH loss at D) 2 months (ANOVA genotype F(1,86) = 50, *P* < 0.001, BSO F(3,86) = 0.4, *P* = 0.75) and E) 11 months (ANOVA genotype F(1,106) = 403, *P* < 0.001, BSO F(3,106)=2.7, *P* = 0.051), but decline gradually at F) 21 month (ANOVA genotype F(1,112) = 208, *P* < 0.0001, BSO F(3,112) = 15, *P* < 0.0001). In significantly different 3xTg-AD neurons, decrements in GSH cause small reductions in NAD(P)H levels at all ages. Tight linear fits indicate direct relationships of NAD(P)H to GSH, but NAD(P)H concentration as a function of extrapolated zero GSH levels in neurons from G) 2-month mice non-Tg slope = −0.0002, intercept 92 μm NAD(P)H (R^2^ = 0.97); 3xTg-AD slope = 0.0002, intercept 33 μm NAD(P)H (R^2^ = 0.92), H) 11-month non-Tg slope = 0.00, intercept 105 μm NAD(P)H (R^2^ = 0.01); 3xTg-AD slope = 0.0002, intercept 39 μm NAD(P)H (R^2^ = 0.99), and I) 21-month non-Tg slope = 0.0003, intercept 36 μm NAD(P)H (R^2^ = 0.95; 3xTg-AD slope = 0.0003, intercept 7 μm NAD(P)H (R^2^ = 0.95). Extrapolations onto the *y*-axis for total depletion of GSH suggests that viable neurons can be depleted of GSH but not NAD(P)H. *n* = 16–20 neurons from 3–4 animals per age per genotype.

For completeness, we determined whether GSH levels reciprocally controls NAD(P)H concentrations, that is, a tight equilibrium, by inhibition of GSH synthesis at the rate-limiting enzyme for GSH synthesis, γ-glutamyl cysteine synthase, with buthionine sulfoximine (BSO) (Ghosh *et al*., 2014). As before, BSO dose-dependently depleted GSH at all ages examined and in both genotypes to similar degrees, although uninhibited levels of GSH were significantly lower in the 3xTg-AD than the non-Tg neurons as before (Fig. 4A–C).

Next, we determined the effect of GSH loss on NAD(P)H concentrations. Considering non-Tg neurons first, with loss of GSH, the 2-month non-Tg neurons increased their NAD(P)H concentration from 63 μm to 74 μm (Fig. 4D), suggesting a compensatory increase in NAD(P)H levels when GSH is limiting. In the 11-month middle-aged non-Tg neurons (Fig. 4E), decreased GSH did not affect the NAD(P)H concentration. It was not until the oldest 21-month non-Tg neurons that we observed a decline of 30% in NAD(P)H concentration with lower GSH at 10 μm BSO. On the other hand, at all ages, the 3xTg-AD neurons behaved more like old non-Tg neurons with NAD(P)H levels, but the percentage decline in NAD(P)H with GSH loss grew larger with age from 26% at 2 month to 62% at 21 month (Fig. 4F).

To further investigate whether NAD(P)H is dependent on GSH, we extrapolated to virtual exhaustion of GSH and correlated NAD(P)H as a function of GSH loss (Fig. 4G–I). Our results for non-Tg neurons reflected negative or no dependence of NAD(P)H on GSH levels at 2 and 11 months, respectively. It was not until 21 months that non-Tg NAD(P)H levels declined with GSH depletion. The 3xTg-AD on the other hand had a non-Tg old-age-like correlation at all ages with slope dNAD(P)H/dGSH remaining nearly constant at all ages. Extrapolating to zero GSH decreased NAD(P)H levels to 37 μm at 2 months and a similar 41 μm at 11 months, ~60% and 40% of the levels of non-Tg neurons, respectively. This indicates that the AD-like neurons lacked the ability to maintain NAD(P)H that was consumed with decrements in GSH, even from the earliest age. At 21 months, extrapolation indicates that 3xTg-AD neurons decreased NADH with GSH depletion in the same proportions as non-Tg neurons, but starting at lower levels of only 40 μm NAD(P)H to levels of only 10 μm. Again, after GSH depletion, significant NAD(P)H remains for all genotypes and ages, indicating that NAD(P)H is not downstream of GSH.

### Age-related neurodegeneration with GSH depletion

**Figure 5:**
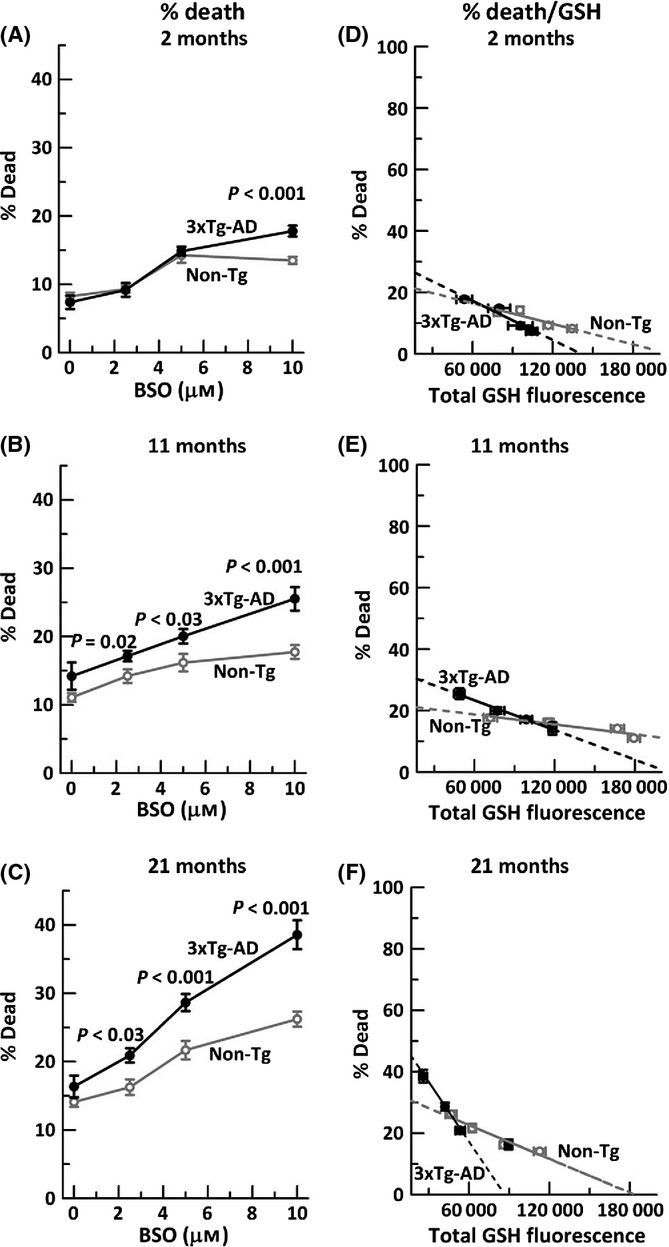
Neurodegeneration due to glutathione depletion increases gradually with age, more so in 3xTg-AD neurons. Glutathione depletion with indicated BSO stress in A) 2-month neurons, (ANOVA genotype F(1,139) = 4.4, *P* = 0.04, BSO F(3,139) = 57, *P* < 0.001), B) 11-month neurons (ANOVA genotype F(1,142) = 26, *P* < 0.001, BSO F(3,142) = 18, *P* < 0.001), and C) 21-month neurons (ANOVA genotype F(1,143) = 22, *P* < 0.001, BSO F(3,143) = 51, *P* < 0.001). *n* = 250–300 neurons from 3–4 mice per age per genotype. Neuron death with GSH depletion and extrapolation to zero GSH levels in neurons from D) 2-month non-Tg mice slope = −0.0001 (R^2^ = 0.83); 3xTg-AD slope = −0.0002 (R^2^ = 0.97), E) 11-month non-Tg slope = −0.00005 (R^2^ = 0.87), 3xTg-AD slope = −0.0001 (R^2^ = 0.99) and F) 21-month non-Tg slope = −0.0001 (R^2^ = 0.94), 3xTg-AD slope = −0.0003 (R^2^ = 0.85). *n* = 3–4 animals per age per genotype.

As glutathione depletion leads to oxidative redox stress and neurodegeneration in both aging and 3xTg-AD neurons (Ghosh *et al*., 2012), we wanted to test whether greater neurodegeneration was caused by depletion of NAD(P)H or GSH. As before (Ghosh *et al*., 2014), we depleted GSH levels by titrating the rate-limiting enzyme glutathione cysteine ligase with BSO (Fig. 5 A–C). We confirmed previous findings that 3xTg-AD neurons are more dependent on GSH availability (steeper slopes) than the non-Tg neurons and that both genotypes accelerated their dependence of neuron loss with GSH decrements with age. The linear relationship between GSH loss and neurodegeneration (Fig. 5 D–F) suggests a simple direct dependence. By extrapolation to zero GSH, the non-Tg cell death increased with age from 21% to 30%, while the 3xTg-AD increased more dramatically from 28 to 45%. Next, we compared these levels of neurodegeneration by GSH depletion to those by NAD(P)H depletion.

### Neuron loss in old age is more sensitive to NAD(P)H loss than GSH loss

To establish the relative importance of NAD(P)H and GSH in aging and neuron loss, we compared 21-month neuron viability extrapolated to zero GSH (Fig. 5F) with those extrapolated to zero NAD(P)H (Fig. 3F) to determine which had a greater effect on neuron loss (Table 2). When GSH or NAD(P)H levels were extrapolated to zero, the % dead for both non-Tg and 3xTg-AD neurons was about twofold greater for NAD(P)H than GSH, indicating that decrements in NAD(P)H are more important to neurodegeneration than GSH. Further, the rate of approach to maximal neurodegeneration (dDeath/dGSH or dNAD(P)H) was also 1.8- to 2-fold greater for NAD(P)H than GSH (Table 2). In addition, these metrics were more severe for the 3xTg-AD than the non-Tg neurons.

**Table 2:** More neurodegeneration by NAD(P)H depletion than GSH depletion*

	21 month	
	Non-Tg	3xTg-AD
% Death intercept at 0 GSH	30%	45%
% Death intercept at 0 NAD(P)H	60%	80%
dDeath/dGSH	−0.3	−0.9
dDeath/dNAD(P)H	−0.6	−1.6

* Change in death with GSH depletion (ddeath/dGSH) was 30%/100% = −0.3 for non-Tg and 45%/50% = −0.9 for 3xTg-AD. Change in death with NAD(P)H depletion (ddeath/dNAD(P)H) was 60%/100% = −0.6 for non-Tg and 80%/50% = −1.6 for 3xTg-AD.

### NADP/NADPH redox state in brain declines with age

**Figure 6:**
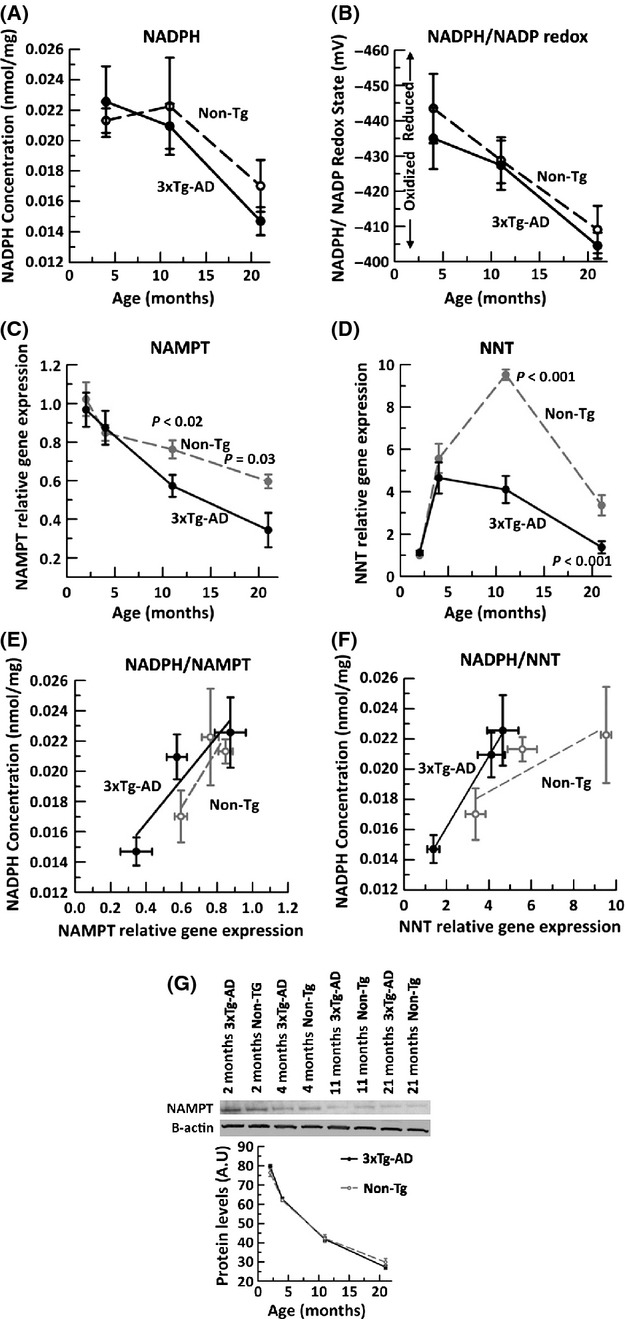
Brain NADPH concentration and NADPH/NADP redox state decline with age and correlate with decline in brain expression of NAMPT and NNT gene expression. By HPLC analysis of cortical/hippocampal tissue homogenates from 4-, 11- and 21-month non-Tg (dashed line) and 3xTg-AD (solid line), A) NADPH concentration (nmol/mg) declines with age (ANOVA F(2,24) = 10.4, *P* = 0.001). B) Calculated NADPH/NADP redox state (mV) using the Nernst equation indicates a large oxidative shift in both non-Tg and 3xTg-AD brain with age (ANOVA F(2,24) = 15.7, *P* = 0.001). qRT PCR on non-Tg (gray filled circle, dashed line) and 3xTg-AD (black filled circle, solid line) brains indicate an age- and AD-related loss in gene expression of metabolic enzymes C) NAMPT (ANOVA, age F(3,33) = 21.8, *P* < 0.001, genotype F(1,33)=6.6, *P* = 0.02), and D) NNT (ANOVA age F(3,33) = 64.08, *P* < 0.001, genotype F(1,33) = 32.417, *P* < 0.001). Fold change expressed relative to 2 month non-Tg GAPDH as internal control. Brain NADPH levels correlate with E) NAMPT (non-Tg R^2^ = 0.76, 3xTg-AD R^2^ = 0.84) and F) NNT (non-Tg R^2^ = 0.76, 3xTg-AD R^2^ = 0.99). *n* = 4–7 animals per age per genotype. (G) Analysis of NAMPT protein by immunoblot indicates a larger decline with age than for mRNA and no genotype difference (*n* = 4 brains from each mouse).

These culture studies were complemented by *in vivo* measures of the NADPH/NADP redox state of brain tissue homogenates by HPLC from 4-, 11-, and 21-month non-Tg and 3xTg-AD animals. We observed that after middle age (11 month), the NADPH concentration declined similarly in both non-Tg and 3xTg-AD brain (Fig. 6A). Likewise, the redox state of both non-Tg and 3xTg-AD brain was significantly more oxidized with age (Fig. 6B) (ANOVA *P* = 0.001). However, we did not observe any genotype difference, possibly due to blood and glial compensation for neuronal changes. These results indicate that the redox state in the whole aging brain reflects an oxidative shift as seen in the cultured neurons.

### Upstream redox dependent NAMPT and NNT enzyme gene expression declines with aging

One mechanism by which the observed age and AD genotype-dependent redox changes could be imposed is through altered gene expression of upstream enzymes that contribute to NAD(P)H regeneration. The synthesis of NAD(P)H is regulated by several redox-dependent enzymes including nicotinamide phosphoribosyltransferase, mitochondrial NAMPT, and the mitochondrial NADPH-NAD^+^ transhydrogenase, NNT. NNT is involved in NADPH and indirect GSH regeneration and under energetic demand, and NNT can regenerate NADH from NADPH (Yin *et al*., 2012). Gene expression of NAMPT in both non-Tg and 3xTg-AD brains was determined by qPCR. NAMPT expression declined steadily with age (Fig. 6C), a 40% overall decline from 2 to 21 months in non-Tg brains and a larger 60% decline in 3xTg-AD brains (ANOVA genotype *P* = 0.015, age *P* < 0.001).

Examining NNT gene expression (Fig. 6D), the brains of non-Tg mice first increased their NNT levels ninefold from 2 to 11 months at middle age and then dropped 61% at the 21 month age. The 3xTg-AD brain on the other hand failed to increase NNT expression after young ages (4 months) and declined 66% with aging at 21 months, below that of the non-Tg brain. Measures of expression of these genes from neuron cultures correlated similarly with those of brain (data not shown). To further correlate our measures of NADPH with changes in gene expression levels in brain tissue, we plotted both NADPH concentration as a function NAMPT gene expression (Fig. 6E) and NNT gene expression (Fig. 6F). NADPH from Non-Tg and 3xTg-AD brain correlated positively with NAMPT gene expression. Similarly, NADPH concentrations in both genotypes were strongly correlated with NNT gene expression. In contrast, NAMPT expression did not correlate as well with GSH levels (R^2^ = 0.3 and 0.05 for non-Tg and 3xTg-AD, respectively, data not shown). The decline in NAMPT mRNA expression (Fig. 6E) extended to a larger decline in NAMPT protein levels, without a genotype difference (Fig. 6G). Our data suggest that NADPH concentration in brain tissue is highly dependent on NAMPT and NNT gene expression.

## Discussion

Redox couples NADH/NAD^+^, NADPH/NADP, and GSH/GSSG along with thioredoxins are vital for diverse biochemical processes including signal transduction, gene expression, bioenergetics, apoptosis, and others. With aging, GSH and NADH decline (Ghosh *et al*., 2012, 2014; Zhang *et al*., 2012) which could contribute to Parkinson’s and Alzheimer’s disease (Jacobs *et al*., 1985). Although GSH and oxidative stress have received more attention, if NAD(P)H levels largely control GSH levels, ROS, and other signaling processes, then restoration of NAD(P)H deficits may be more important and productive. Here, we show that between NAD(P)H and GSH, NAD(P)H is more important for neuron survival in aging and Alzheimer’s disease model neurons, in particular. Moreover, we observed that upstream redox enzymes NAMPT and NNT positively correlate with brain NADPH in non-Tg and 3xTg-AD mice. The failure of recent antioxidant human trials and the failure of certain antioxidant transgenic mice to extend lifespan (Perez *et al*., 2009) could be due to ignoring a more seminal cause of NAD(P)H deficits.

NADH, the reduced form of NAD^+^, is not only the major electron carrier down the electron transport chain, but also vital for cellular signaling in neurons through calcium homoeostasis (Kaplin *et al*., 1996), mitochondrial permeability transition (MPT) (Chernyak & Bernardi, 1996), apoptosis (Kahraman & Fiskum, 2007), and gene transcription regulation (Fjeld *et al*., 2003). Our observed decreased NAD(P)H and redox ratio with aging and AD may be due to declines in TCA cycle enzymes that generate NADH including pyruvate dehydrogenase, isocitrate dehydrogenase, alpha-ketoglutarate dehydrogenase, and malate dehydrogenase (Bubber *et al*., 2005; Yao *et al*., 2009). With the help of intrinsic autofluorescence of NAD(P)H, we observed that in younger non-Tg neurons until middle age, NAD(P)H adjustments can compensate for GSH loss indicating its upstream redox control. In fact, Garcia *et al*. (2010) observed that adding glutamate/malate to mitochondria to power the shuttle of electrons into NADH in the mitochondria increased the GSH concentration and simultaneously decreased GSSG, supporting our observation that NAD(P)H redox control is upstream of GSH redox control.

Our present observation of a higher neuron loss as a function of NAD(P)H loss associated with aging and AD may be due to the following factors. (i) A more oxidized NADH/NAD^+^ redox ratio opens the mitochondrial permeability transition pore, causing mitochondrial depolarization, loss of energy-generating capacity and cell death by apoptosis and/or necrosis (Kahraman & Fiskum, 2007), and lower expression of anti-apoptotic gene bcl-2 (Ellerby *et al*., 1996). (ii) The apoptosis inducing factor (AIF) is an NADH oxidase so that an oxidative shift in NADH/NAD^+^ ratio will induce apoptosis in a caspase-independent pathway (Guarente, 2011). (iii) The oxidized product of NADH-dependent redox reactions, NAD^+^, serves as a cofactor for sirtuins that promote longevity in several organisms including yeast, worms, and rodents (Guarente, 2011) so that a decline in NAD^+^ (without sufficient regeneration by dehydrogenases) would lower sirtuin activity, promoting neurodegeneration. Our demonstrated ability to improve neurotoxic resistance with the NAD^+^ precursor, nicotinamide (Ghosh *et al*., 2014), further supports this proposition. (iv) A decline in NAD with aging would lead to failure of the NAD-dependent enzyme poly-ADP-ribose polymerase-1 (PARP-1) to repair ssDNA breaks that accumulate with oxyradical stress (Diaz-Hernandez *et al*., 2007), contributing to genomic instability and cell death. e) On the other hand, the greater cell death in our 21-month 3xTg-AD compared with non-Tg neurons could result from over activation of PARP-1 by both reactive oxygen species and beta-amyloid, consuming even more NAD to promote AIF-mediated apoptotic neuronal death (Strosznajder *et al*., 2012). Thus, loss of NAD(P)H will lead to an energy crisis and pleiotropic induction of risk factors for increased neurodegeneration in aging and AD.

The dependence of GSH on NADPH-associated glutathione reductase is well known, but the relationship of NAD(P)H to 10-fold higher NADH levels is less clear. We and others have observed an age- and AD-related decline in GSH (Ghosh *et al*., 2012, 2014; Zhang *et al*., 2012); however, several attempts to increase GSH levels by overexpressing γ-glutamyl cysteine synthetase (Gclc) or GR have failed to increase longevity (Perez *et al*., 2009) or improve memory. A plausible explanation for this failure may be that the GSH redox system is downstream of a more important NAD(P)H redox couple. The inability of GSH to increase NAD(P)H levels when stressed strongly suggests that GSH redox system is downstream of the NAD(P)H redox system. However, when GSH is limiting, other antioxidant/reducing substrates such as superoxide dismutase, catalase, and thioredoxins may still provide enough protection against degeneration to compensate for GSH loss (Ghosh *et al*., 2014). This would explain our observation of less neuron loss with GSH depletion than with NAD(P)H depletion in aging and AD. The early GSH decline in 3xTg-AD neurons is supported by declines in glutathione S-transferase and glutathione peroxidase in human AD (Ansari & Scheff, 2010). Further, our extrapolations to limiting NAD(P)H and GSH show that GSH can be depleted before NAD(P)H and that short-term viability requires minimal NAD(P)H levels. Most importantly, substrates such as malate and NADH (Garcia *et al*., 2010) can generate NADPH through nicotinamide nucleotide transhydrogenase, which in turn can recycle GSH thorough glutathione reductase, putting GSH downstream of NADH redox system.

Both NAD(P)H and GSH are controlled by several upstream enzymes such as NAMPT, NNT, and Kreb’s cycle dehydrogenases. Titrating with BSO to deplete GSH did not affect NAD(P)H concentrations at younger ages of non-Tg, perhaps due to sufficient activity of Kreb’s cycle dehydrogenases and the pentose phosphate cycle glucose-6-phosphate dehydrogenase to generate NADH and NADPH, respectively, along with the salvage pathway from nicotinamide and *de novo* synthesis of NAD(P)H from tryptophan at younger ages. In the AD brain, α-ketoglutarate dehydrogenase, isocitrate dehydrogenase, and malate dehydrogenase from the TCA cycle all decline (Bubber *et al*., 2005). These deficits would decrease NADH generation and could be responsible for the lower NAD(P)H seen in our 3xTg-AD neurons. In 3xTg-AD mice, pyruvate dehydrogenase and cytochrome oxidase enzyme activity decline even more than the non-Tg (Yao *et al*., 2009) which support the age and AD-related decline in NAD(P)H we observed. The protein levels of the rate-limiting enzyme for NAD synthesis, NAMPT also decline in the hippocampus of old mice compared with young ones (Liu *et al*., 2012) which is consistent with our gene expression studies. A decline in NAMPT would promote inflammation, apoptosis, and less availability of NAD^+^ for sirtuin activity.

Another important upstream redox-sensitive enzyme is NNT, which we observed to decline after middle age in non-Tg but earlier in the 3xTg-AD mice. NNT is a mitochondrial inner membrane transhydrogenase that typically transfers a hydride from NADH to form NADPH, which is then utilized to regenerate GSH from GSSG through GR. However, if there are energetic deficits, NADH can be generated from NAD^+^ via NNT in a redox-dependent manner. NNT mutation or deletion impairs mitochondrial function (Yin *et al*., 2012) and can lead to glucose intolerance and insulin resistance (Freeman *et al*., 2006), both implicated in AD. 3xTg-AD mice are also glucose intolerant (Nicholson *et al*., 2010) which could be part of a vicious cycle of redox imbalance, insulin resistance, and decreased energy-generating capacity, part of the EORS theory of aging (Brewer, 2010; Ghosh *et al*., 2012). As NADPH and NNT are tightly coupled, a decline in NADH and NADPH would also decrease GSH. Our results indicate that brain hippocampal NAMPT and NNT both correlate with NADPH levels. These high correlations suggest tight coupling at the gene expression level to control redox state and that these controls shift toward an oxidized state due to age-related environmental signals, the most important of which could be a sedentary lifestyle. We plan to systematically manipulate NAMPT and NNT gene expression.

Aging is accompanied by a sedentary lifestyle with low physical and mental exertion with consequential bioenergetic decrements (Safdar *et al*., 2010). With lack of activity, energetic demands decrease in neurons, which decrease the need for NADH generating capacity to make ATP. According to our epigenetic oxidative redox shift (EORS) theory (Brewer, 2010), this metabolic stress down-regulates redox enzymes resulting in a vicious and catastrophic cycle of an oxidative redox shift, imposed by epigenetic controls (Walker *et al*., 2012) and further declines in redox buffers NADH/NADPH and GSH. Moreover, in AD, deposition of A-beta, inflammation, and apoptosis would further increase oxidative damage, creating a vicious cycle of neurodegeneration.

Our results here indicate the importance of redox buffers for neuron survival. In fact, we recently showed that in old non-Tg and 3xTg-AD neurons, adding an NAD(H) precursor, nicotinamide along with a Nrf2 inducer, 18α glycyrrhetinic acid, to stimulate a balanced redox defense is more neuroprotective than either alone (Ghosh *et al*., 2014). The net depletion of NAD(P)H in 21-month non-Tg and all ages of 3xTg-AD neurons is caused by deficits in NAD(P)H regenerating capacity in these transgenic mice (Ghosh *et al*., 2012). Targeting the upstream NADH may prove more effective than downstream GSH or ROS for future longevity and neurodegenerative therapies.

## Experimental procedure

### Mouse model and cultured neurons

The use of the 3xTg-AD mouse (Oddo *et al*., 2003), husbandry and genotyping were previously described (Ghosh *et al*., 2012). Adult neurons from the hippocampus and frontal cortex were isolated from non-Tg and 3xTg-AD age-matched male mice at 2 (young), 11 (middle age), and 21 (old)-month time points as before (Brewer & Torricelli, 2007; Ghosh *et al*., 2012).

### Buthionine sulfoximine (BSO) titration or NAMPT inhibition followed by NAD(P)H, FAD, GSH, and ROS measurements in live neurons

At 8 days in culture, 100% of the media containing antioxidants in the B27 was replaced with Neurobasal A/0.5 mm Glutamax. For glutathione inhibition, indicated concentrations of L-BSO (Sigma-Aldrich #B1525, St. Louis, MO, USA) were added to the cultured neurons in 0.01% DMSO vehicle and incubated for 15 hrs at 37 °C in 5% CO_2_, 9% O_2_ at saturated humidity. To deplete NAD(P)H, neurons were treated for 15–16 h with 10 nm FK866 (Sigma-Aldrich) prepared in Neurobasal A, as a specific inhibitor for NAMPT (Wang *et al*., 2011). Single live cells were imaged for intrinsic NAD(P)H and fluorescence as before with excitation at 350 nm and emission at 450 nm (Ghosh *et al*., 2012). Live ROS and GSH levels were determined using dichlorofluorescein diacetate and monochlorobimane and (Brewer *et al*., 2010). Nonfluorescent monochlorobimane is readily taken up by cells and forms a fluorescent adduct GSH-monochlorobimane, catalyzed by glutathione S-transferase in proportion to GSH levels, that can be measured as specific fluorescence (Kamencic *et al*., 2000).

### HPLC of adenine nucleotides and brain thiols

Extraction, derivatization, and analysis of brain NADP/NADPH was performed as before (Ghosh *et al*., 2012). Anesthetized animals were decapitated into liquid nitrogen to rapidly quench metabolism. Approximately 0.04 g of combined hippocampus and cortex was dissected in Hibernate A at 4 °C. The tissue was homogenized, stabilized, and subjected to HPLC with fluorescence detection. We used the Nernst equation to calculate the redox state of the tissue, E_h_ = E_0_ − 2.3 (RT/nF)log[(NADPH)/(NADP)], with the standard potential, E_0_ = −370 mV. HPLC for brain GSH and GSSG was performed as before (Ghosh *et al*., 2014).

### RNA isolation and RTqPCR for Nnt and Nampt gene expression

Flash frozen hippocampus and cortex weighing ~ 30 mg (±10 mg) were extracted in 1.0 mL QIAzol lysis reagent (from the RNeasy Lipid Tissue Mini Kit; Qiagen # 74104, Valencia, CA, USA) and homogenized. The manufacturer’s protocol yielded ~20 μg RNA. One microgram of total RNA was used to create a cDNA pool of ~30 μg utilizing the High Capacity RNA-to-cDNA Kit (Applied Biosystems, #4387406, San Francisco, CA, USA). For qPCR, 60 ng of cDNA was reacted in 20 μL with probe + primers for mouse Nnt (Mm01298456_m1 Nnt, Cat. # 4331182) or Nampt (Mm00451938_m1 Nampt, Cat. # 4331182) in a 2 × master mix (Applied Biosystems # 4369016) in an AB StepOne Plus PCR system (Applied Biosystems) for 10 min at 95 °C for enzyme activation followed by 40 cycles of 15 s denaturation at 95 °C and 1 min anneal/extend at 60 °C. The StepOne Software v2.1 (Applied Biosystems) determined the cycle threshold (Ct). The relative change in mRNA levels between untreated control (i) and treated sample (ii) was measured using the following formula normalized to the levels of GAPDH: 2^(Ct gene1-Ct GAPDH1)-(Ct gene2-Ct GAPDH2) (Soong *et al*., 2001).

### Live–dead assay and statistics

For neuron survival, live cells on glass coverslips were stained with fluorescein diacetate (15 μg/mL; Sigma–Aldrich) and dead cells with propidium iodide (4.6 μg/mL; Sigma–Aldrich). After washing the slips with HBSS (Invitrogen, Carlsbad, CA, USA), cells were observed by blue and green fluorescence excitation through a 20× objective (Olympus, Center Valley, PA, USA) for green (live) and red (dead) fluorescence. Wash solutions were also collected for released dead cells and added to the adherent dead cell count. Percent dead cells was calculated as the average percent dead divided by the total cells (live + dead) in 5–8 adjacent 20× fields. Data are presented as means and standard errors. Student’s *t*-test was used to assess the difference of means using Prostat (Poly Software, Pearl River, NY, USA). We used *P* < 0.05 to reject the null hypothesis. Two-way ANOVA with replicates was performed as indicated.
